# The fine line between automation and augmentation in website usability evaluation

**DOI:** 10.1038/s41598-024-59616-0

**Published:** 2024-05-02

**Authors:** Andrea Esposito, Giuseppe Desolda, Rosa Lanzilotti

**Affiliations:** Department of Computer Science, University of Bari Aldo Moro, Via E. Orabona 4, 70125 Bari, Italy

**Keywords:** Computer science, Information technology

## Abstract

Artificial Intelligence (AI) systems are becoming widespread in all aspects of society, bringing benefits to the whole economy. There is a growing understanding of the potential benefits and risks of this type of technology. While the benefits are more efficient decision processes and industrial productivity, the risks may include a potential progressive disengagement of human beings in crucial aspects of decision-making. In this respect, a new perspective is emerging that aims at reconsidering the centrality of human beings while reaping the benefits of AI systems to augment rather than replace professional skills: Human-Centred AI (HCAI) is a novel framework that posits that high levels of human control do not contradict high levels of computer automation. In this paper, we investigate the two antipodes, *automation vs augmentation*, in the context of website usability evaluation. Specifically, we have analyzed whether the level of automation provided by a tool for semi-automatic usability evaluation can support evaluators in identifying usability problems. Three different visualizations, each one corresponding to a different level of automation, ranging from a full-automation approach to an augmentation approach, were compared in an experimental study. We found that a fully automated approach could help evaluators detect a significant number of medium and high-severity usability problems, which are the most critical in a software system; however, it also emerged that it was possible to detect more low-severity usability problems using one of the augmented approaches proposed in this paper.

## Introduction

Recent technological advances have enabled the development of novel Artificial Intelligence (AI) machines that automate most tasks: mortgage calculators^1^, medical diagnosis^2^, and art generation^3^, to mention but a few. However, AI’s increasing pervasiveness has raised concern over the existing techniques’ flaws. Among these, biases and lack of explainability endanger the users of the AI models, which often do not consider the human element^4^. In 2016, a report by the National Transportation Safety Board regarding a deadly crash of an autonomous Tesla car stated the following: «automation “because we can” does not necessarily make the human-automation system work better. […] This crash is an example of what can happen when automation is introduced “because we can” without adequate consideration of the human element»^5^. With these words, the report wanted to highlight the importance of the human element in the interaction between the user and the intelligent system.

AI models can automate or augment user tasks to improve efficiency, accuracy, and overall user experience (UX)^6^. Automation is used to perform repetitive or time-consuming tasks, freeing up users’ time for more important tasks. Examples are AI models that automate repetitive administrative tasks such as data entry, invoicing, or scheduling. As a more complex example, consider DALL-E, an AI system to generate images: the system receives a prompt describing what the user wants (e.g. “draw a cat in the style of Monet”) and uses it to generate images^3^. Although users have granular control over the prompt (as they are the ones who write it), once the input is received, the AI automatically generates its output, and users have no control over the generation process itself. Conversely, augmentation can be used to enhance human capabilities and decision-making by providing additional information, insights, or recommendations. An example of augmentation is an AI model that augments a doctor’s diagnosis by providing additional medical information, such as possible diagnoses or treatment options, or an AI model that augments a financial analyst’s work by providing real-time financial data and market insights to inform investment decisions.

In the novel field of Human-Centred Artificial Intelligence (HCAI), one of the main goals is to provide techniques to produce AI systems that are Reliable, Safe, and Trustworthy^7^. To reach this goal, AI systems should provide a high level of computer automation while guaranteeing a high level of human control when desired^6^. In other words, AI systems should not *automate* tasks but amplify, *augment*, empower, and enhance their users’ capabilities^8^. The right level of automation or augmentation depends on the specific task, the user’s needs and preferences, and the overall context of the task and must be designed accordingly.

In this research, we explore the two antipodes, *automation vs augmentation*, in the context of website usability evaluation. Although documented benefits of usability evaluation methods exist, still today, too many companies and practitioners neglect them for two main essential reasons. First, it is generally agreed that usability experts are a scarce resource^9^. In addition, companies complain that usability methods are resource-demanding and that no methods that suit companies’ needs exist^10^. However, it is widely recognized that usability evaluations improve the overall quality of their products^11^. Automatic or semi-automatic tools could assist evaluators with inadequate skills in performing reliable usability evaluations. In this way, usability evaluations can better meet companies’ needs.

To overcome these misconceptions that limit the spread of usability culture, we explored AI as a way to simplify usability studies. The ground of this study is SERENE, a web platform for semi-automatic ﻿UX evaluation of websites^12,13^. It uses an AI model based on neural networks to predict visitors’ emotions based on their interaction logs. The concentration of negative emotions in a specific area of the website can help evaluators identify UX problems^14,15^. This platform is in the range of solutions that (partially or totally) automate usability evaluation^16,17^. However, the study of the right level of automation and augmentation that favours the quality of the usability evaluation has never been considered in this field. For example, automating the discovery of usability problems would be a panacea for the Human-Computer Interaction (HCI) field. However, we may be very far from this because, despite the precision of the AI solutions adopted, manual intervention by usability experts is still needed to avoid false positives and false negatives and because usability depends on the tasks, users, and context^18^; thus a fully automated approach may not fit all these different dimensions. On the contrary, providing evaluators with an augmentation technique that presents too much information about the website visitors, may require technical skills to interpret such data, cause cognitive overload, and ultimately determine tool abandonment. In other words, varying the level of automation affects users’ bias, causing or preventing errors due to a loss in users’ attention^19–21^.

In this research, we have extended SERENE to understand the differences between different levels of automation and control, ranging from a fully automated approach (i.e., the system lists the usability problems) to an augmented approach (i.e., the system leaves the decisions to the evaluators), including an intermediate approach. With this research, we aim to answer the research question “*How does the level of automation affect the identification of usability problems?*”. The contributions of this paper are the following:

- Three different visualizations to report the usability problems, each one corresponding to a specific level of automation/control;
- An experimental comparison to understand how the different levels of automation/control affect the identification of usability errors in terms of numerosity and severity, evaluating the number of errors due to automation bias;
- Lessons learned on the different levels of automation or augmentation approaches.

The paper is structured as follows. The next section introduces the context of this study, presenting the platform under evaluation and the different approaches to reporting usability problems. Then, the third section details the methodology used to conduct the study and collect the data. The fourth section presents the results of the experiment, which are then discussed in the fifth section, followed by the identification of potential threats to the validity of this study. Conclusion and future work close the paper.

### The SERENE platform and the different levels of automation and augmentation

In the last years, there is a growing proliferation of solutions that (partially or totally) automate usability evaluation^16,17^, typically by measuring users’ emotions and detecting usability problems when negative emotions are felt during the interaction with the evaluated systems^14,15^. In general, we were able to identify two trends in this regard. Some authors apply techniques of affective computing to recognize emotions using data from interaction logs^16,17,22^. However, such works do not connect emotions and usability. Others, instead, aim at providing static analyzers for usability problems. A review of such systems by Namoun et al.^23^ highlights the main problems of such systems. One of them is that most of these systems provide their scores as an overall percentage or a rank (from A to F), increasing the difficulty in identifying the actual usability problems^23^.

The SERENE platform aims at joining the two trends by providing a way of detecting actual usability problems^13^. It measures the emotions website visitors feel (the seven emotions of the Ekman model are used^24^) while browsing a website, without requiring any software or hardware on their device. Specifically, the user’s mouse and keyboard keystrokes are recorded via a JavaScript file installed on the website^12^. This data is then converted into emotions thanks to an AI model based on a neural network. One of the peculiarities and novelties of SERENE is that it predicts the user’s emotions moment by moment during the user’s interaction. In this way, by aggregating all user logs, it is possible to predict the average emotions website visitors feel while interacting with each user interface (UI) element. SERENE uses the information about emotions to suggest to its users (usability evaluators) where potential usability problems can be found on each web page^13^. This is based on the assumption that UI elements (e.g. menu, labels, images, widgets) affected by usability problems cause negative emotions^15^. For example, the results of a study performed by Seo et al.^25^ revealed that perceived usability is positively correlated with emotional valence and negatively correlated with emotional engagement. Similarly, in the context of online platforms, different studies investigated the emotional and cognitive state while evaluating the usability and UX of an online course platform, demonstrating that emotional responses can influence how users perceive the usability of e-learning platforms^26,27^. Furthermore, Zakaria et al.^28^ found a high correlation between mobile app usability and positive emotions, highlighting the impact of emotions on user experience.

To report the usability problems, and given the purpose of the study, we created three different visualizations, each corresponding to a different level of automation and control.

**Figure 1:**
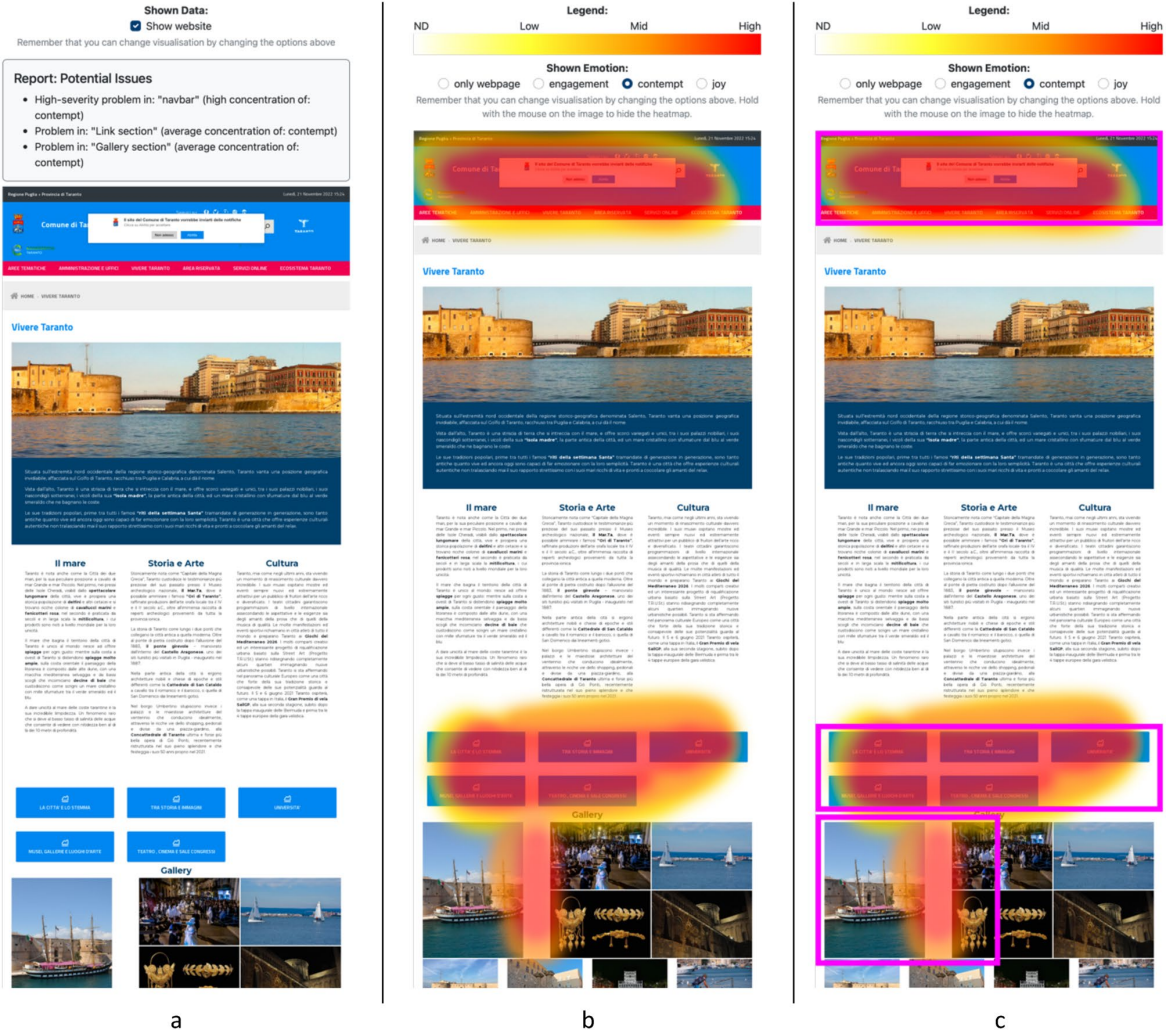
The different levels of automation/augmentation implemented in SERENE to help discover usability problems: (**a**) a list of the usability problems; (**b**) a heatmap showing the concentration of the emotions overlapped to the webpage; (**c**) the heatmap of the previous visualization also extended with purple rectangles highlighting potential usability problems.

The first one is called *full automation* and shows, for each webpage, the textual list of user interface elements potentially affected by usability problems. In addition, to help the evaluators’ analysis, the related website is reported below it (Fig. 1a). Areas with content where negative emotions exceeded a certain threshold were considered to have usability problems. The threshold was set empirically by conducting usability studies with both real usability evaluators and SERENE on the same websites; this allowed us to determine, for each emotion, the threshold at which SERENE acted as a usability expert.

The second, called *full augmentation*, shows, for each web page, a heatmap overlaid on that page. Above it, there is a menu that can switch between the 3 emotions and a legend that shows the interpretation of the emotion intensity (Fig. 1b). The emotions that the expert can analyze are contempt, joy, and engagement. The most important is contempt, which is a negative emotion felt by visitors and symptomatic of usability problems^14,16,29–32^. Joy has been included because it reveals parts of the website that may not be affected by negative emotions and, therefore, not usability problems. On the other hand, engagement is obtained by composing all 7 emotions and, in this context, indicates how the website elements engaged visitors. In this case, nothing is explicitly reported about the usability problems. Still, the evaluators have to freely interpret the concentration of emotions on the user interface elements, thanks to the heat map, to determine usability problems, for example, where a high concentration of contempt is visualized. Participants could still choose the visible emotion and the ability to hide the heatmaps.

The last visualization is called *middle-ground*: it shows the same information as in the case of full augmentation, but the visualization is also extended with purple rectangles that highlight potential usability problems (e.g., due to a high concentration of negative emotions). This condition can still be considered an augmentation technique, as users are still in control of their decisions, but they could be helped by the presence of such rectangles, which aim to facilitate the identification of usability problems (Fig. 1c).

## Method

This section reports the experimental study conducted to understand the right level of automation/augmentation (automation for short in the following) a system, the SERENE platform, should provide to adequately support evaluators in discovering usability problems. It is worth noting that there is no intent to evaluate the SERENE system itself; it has been used since it well represents the class of solutions for automating usability tests.

The experimental evaluation was designed as a survey and consisted of a questionnaire administered on a web platform that asked participants to evaluate two websites. In this evaluation activity, participants have been supported by the three different SERENE solutions providing different levels of automation.

### Design

A between-subject design was adopted. The level of automation was the independent variable. The three between-subject levels are *full automation (*Fig. 1a), *full augmentation* (Fig. 1b), and *middle-ground* (Fig. 1c). The study’s goal is expressed more formally by the following research question: “*How does the level of automation affect the identification of usability problems?*”. To study this RQ, we identified the following null hypotheses:

- H_01_: The number of usability problems identified by the evaluators does not depend on the level of automation.
- H_02_: The perceived severity of usability problems identified by the evaluators does not depend on the level of automation.

Eight metrics were defined to address the research question and verify the hypotheses. Specifically M1, M2, M3, and M4 relate to H_01_, while M5, M6, M7, and M8 relate to H_02_. In the following, we report the formula of each metric detailed with a brief explanation:

- *M*1 = *the number of usability problems identified by the evaluator and shown in the visualization*. It serves as an indicator of the solution’s effectiveness in facilitating the identification of usability problems.
- *M*2 = *the number of false positives identified by the evaluator and shown in the visualization.* False positives have been purposely introduced in this study because, in real scenarios, they may result from incorrect classification by the AI model being used; this metric reflects the ability of the report to assist evaluators in avoiding the identification of false positive usability problems. This metric measures the number of *commission errors* by the evaluators due to automation bias^19^. In this study, only a single false positive was introduced, therefore the metric shows whether the visualization induced a commission error or not.
- *M*3 = the *number of usability problems discovered by evaluators but not reported in the visualization.* The AI models may not detect some usability problems; therefore, to account for this situation that may occur in real scenarios, we do not consider some usability problems in the managed visualisations; this metric provides insight into the extent to which the report does not hinder the detection of problems not explicitly highlighted in it.
- *M*4 = *the number of usability problems not discovered by evaluators because not reported in the visualization*. This metric measures the number of *omission errors* by the evaluators due to automation bias^19^.
- *M*5 = ${\left|{E}_{i}\right|}^{-1}\cdot {\sum }_{e\in {E}_{i}}{\left({\text{severit}}{\text{y}}_{\text{evaluator}}\left(e\right)-{\text{severit}}{\text{y}}_{\text{expert}}\left(e\right)\right)}^{2}, {E}_{i}\text{ set of errors identified by evaluator}\,i.$ This metric refers to the ability of the visualization in communicating the severity of usability problems. In this study, errors were categorized into three levels of severity (low, medium, and high) by participants. Therefore, each error correctly identified by a participant may deviate by one or two levels from the proper severity level as determined by the authors of this article.
- *M*6, * M*7, and * M*8 are the number of usability problems identified by the evaluator of high, medium, and low severity, respectively.

### Participants

As inclusion criteria to recruit participants, we have decided that evaluators must have low or medium expertise in usability studies. This was done because automated solutions for usability tests, like the ones proposed in this study, are intended to empower mainly individuals without an extensive background in HCI and usability evaluation to increase the prevalence of usability testing in real contexts. To meet this criterion, we recruited volunteers from the third year of the Bachelor of Science in Computer Science degree program who had successfully completed a course on HCI, which included instruction on evaluation methods such as heuristic evaluation and user testing. We initially recruited 103 participants; then, 4 of them were removed after a quality check of their answers performed by two of the authors of this study (e.g., eliminating participants whose answers were incomplete or technically wrong). Thus, 99 participants (15 females, 84 males) were finally considered (N_full automation_ = 33, N_full augmentation_ = 35, N_middle-ground_ = 31). The collected demographics indicated that the selected participants are on average 22.16 years old (SD: 2.46).

Throughout the data collection, we followed common practices, relevant guidelines, and regulations for studies involving users and respected ethical principles: we obtained informed consent, protected the confidentiality of participants, minimised harm, ensured voluntary participation, and protected their privacy. A retrospective ethical waiver for the experiment was received from the Independent Ethical Committee associated with the University of Bari (protocol number: 0053678|08/06/2023, study number 7772).

### Materials and apparatus

Participants were instructed to evaluate the home page’s usability of two websites: the website of an Italian municipality (https://www.comune.taranto.it/) and the website of an Italian region (https://www.regione.basilicata.it/). These websites were selected following a thorough process of usability evaluations on multiple websites; the presence of usability problems, which are also heterogeneous among them, made them suitable candidates for our study.

An important aspect of this study was the creation of the ground truth, i.e. the list of usability errors identified by the experts on the two websites. The ground truth played a very important role for different reasons. First, since each visualisation does not specify usability problems that affect UI elements, we associated their information (e.g., heatmap depicting the concentration of emotions or textual list of user interface elements potentially affected by usability problems) with the errors in the ground truth. For example, in the case of Fig. 1b, there is a big cluster of contempt emotions in the bar of the webpage https://www.comune.taranto.it/, which we linked with 6 different usability errors of the ground truth. Second, the ground truth allowed us to validate the participants’ reports to discard usability errors that were not real and to standardise the names of usability errors that participants labelled differently. Therefore, this use of the ground truth associated with the visualisations and participant reports acted as a common layer that allowed to compare visualisations with participant reports.

The process of identifying usability errors in the ground truth followed the same method adopted by the participants, namely the heuristic evaluation based on Nielsen’s 10 heuristics. Jakob Nielsen indicates that five novice evaluators can help discover about 75% of the usability issues. However, the number of issues relies on the evaluator’s expertise. Thus, to ensure the identification of the vast majority of usability errors on the administered web pages, three expert evaluators have been involved (the authors of this study) and performed three evaluation cycles, for each of which they first acted individually and then compared with each other. Only three cycles were performed because, by the third cycle, the usability errors found, excluding those already identified in the previous cycles, were very few and no further evaluation cycle was necessary. In the first evaluation phase, the reliability was 70%, in the second cycle 85%, and in the third cycle 95%. At the end, we created a list of 46 usability errors (21 errors for the first website, 25 for the second website), each one associated with a severity rating from 1 to 3 (1 low severity, 3 high severity).

In the case of the first website, different usability problems were identified by the three authors of this study. For example, one such problem was located in the navigation bar at the top of the website, which was found to be excessively tall and covered a significant portion of the screen, thus impeding the page’s visibility. Additionally, in the same area, a pop-up related to cookies obscured some crucial functions, such as the search bar. Another problem was identified in a section containing links to various details about the city, where the designers utilized the same icon to represent different links, potentially confusing users. Furthermore, in the gallery section, some images were observed to have different margins, which, although not a formal usability problem, users may find aesthetically unpleasing. Finally, in the footer, some links were found not to function correctly, and outbound links were found to be mixed with internal links.

Likewise, different usability problems were discovered on the second website by the authors of this study. For example, this website is characterized by a multi-column non-responsive layout (typically found in older websites), making the page seem cluttered and overwhelming. Additionally, the website is riddled with colours and images, which may confuse the users. From top to bottom, the website’s navigation bar is split into multiple bars without a clear reason, and the design and style of the links may confuse users into thinking that they are part of a breadcrumbs sequence. Then, the user is greeted by a banner that does not communicate well, whether it is clickable or not, or if only some parts of it are. The headings of the various sections are unclear and do not clearly communicate the kind of information they group to the user. Another set of problems was identified in the right sidebar: the sheer number of colours and information and the lack of a standard way of presenting content may confuse and overload the users. Moving to the central section, the tab bar of alerts’ categories does not clearly communicate being a legend for the coloured strips associated with each alert. Furthermore, Gestalt’s principle of proximity is violated in the same section as the links to get more information are not always near the title to which they refer^33^. Finally, the website does not provide basic accessibility functions (like font size pickers) and the date of the last update.

For the evaluated websites, three different visualizations, each one corresponding to one of the experimental conditions, were created. Their creation took into account the ground truth, as well as the dependent variables to be measured. In particular, we started from data actually collected by SERENE during the interaction with the administered websites. The resulting visualizations have been then modified to introduce a false positive to measure M2: on the website for the municipality, we highlighted a minor problem with margins between images as a problem, as it seems likely that unbalanced margins may provoke a reaction in users, without it being an actual usability problem. In addition, by comparing the results of SERENE with the ground truth, we made sure that SERENE had failed to detect some usability errors to measure M3: for the website of the municipality, we found that the errors regarding typos and the general overload in the footer, as well as problems regarding the general design of the webpage and minor hard-to-detect problems (e.g., missing update date, missing font size buttons), were not detected by SERENE; for the website of the region, errors regarding the general design and overload of the page and some of its sections, errors regarding the relationship between elements of the page (e.g., unclear ordering of the alerts, the ambiguous relationship between headings and content), as well as minor hard-to-detect problems were not detected. It is worth noting that this study does not aim to compare the errors identified by the experts and those identified by SERENE.

To administer the study, an online web platform was developed using Node.js. The participants were presented with a screen displaying, on the left side, the visualization corresponding to the administered experimental condition and a set of questions on the right side. The first question asked participants if the visualization enabled them to identify usability problems. If so, they were instructed to list all identified problems in a form containing three fields: the UI element affected by the problem, a description of the problem, and a severity rating (on a scale of 1–3, with 3 indicating the highest severity). The second question asked participants if they identified any usability problems that the visualization did not show; similarly to the first question, if the evaluators found some problems, they could report them in the related form. Additionally, a link to the 10 Nielsen Heuristics was provided at the top of the webpage for use by the evaluators, to aid them during the evaluation.

### Procedure

Initially, the participants were invited to the study by email containing the link to the survey platform. Once the participant clicked on the link, a page providing an overview of the study, and the intended use of the participants’ responses appeared. Participants were asked to consent to participating in the study through a digital form prompted before the commencement of the survey. Participants who did not provide consent were allowed to discontinue the survey. All participants provided consent. Once consent was obtained, the platform requested participants’ gender, age, and email addresses. The other data were collected anonymously, with no means of identifying individual participants. The platform randomly assigned participants to one of the experimental conditions. No questions were asked that would reveal the participant’s identity. Participants subsequently performed evaluations of the two websites, one at a time, reporting usability problems in the appropriate forms, if any. After the evaluation, participants were thanked for their participation. The entire procedure and materials were previously assessed in two pilot studies involving 5 and 6 participants, respectively.

### Data analysis

Two of the authors of this study conducted a thorough analysis of all participants’ responses to remove any instances of careless participation or low-quality answers. As previously reported, this process resulted in the exclusion of 4 participants. Subsequently, each response was then carefully evaluated by the same researchers to determine the accuracy of the usability problems reported by the participant, using the ground truth that detailed the actual usability problems present in the websites. In total, 479 usability problems were reported by participants and, according to the ground truth, 391 of these problems were correct, while 88 were incorrect. This study phase required approximately 110 h of effort from each researcher.

Following this, the eight metrics were calculated for each participant. For all the metrics, except M2, the Kruskal–Wallis H-Test was utilized to compare the three experimental conditions because of the violation of normality assessed with the Shapiro–Wilk Test; in case of statistical difference, the Mann–Whitney U Rank Test has been employed as a post-hoc test. In the case of M2, since it is a dichotomous variable (false positive identified or not), the Chi-Square test has been employed. A significance level of 0.05 was considered for all these statistical tests.

## Results

**Figure 2:**
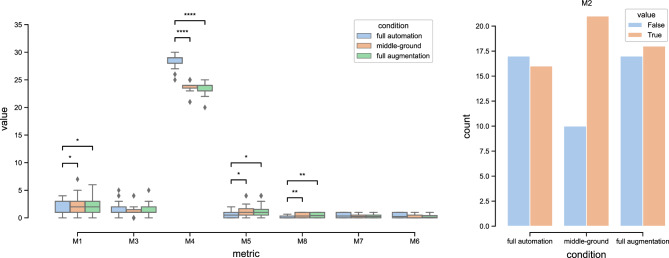
Graphs of the eight metrics with annotated statistical differences.

To investigate the two hypotheses related to the RQ of this study, we compared the three different levels of automation implemented in SERENE along the eight metrics. All the descriptive and inferential statistics details are reported in Table 1. A summary is depicted in Fig. 2.

**Table 1:** Details of the descriptive and inferential statistics.

		Full automation (FA)		Middle-ground (MG)		Full augmentation (AU)			
Metric		$\overline{{\text{x}} }$	s	$\overline{{\text{x}} }$	s	$\overline{{\text{x}} }$	s	Test results	Post-hoc test results
H_1_	M1 (correct classification)	1.485	1.253	2.355	1.582	2.343	1.474	H(2) = 7.373 *p* = 0.025*	FA-MG: U = 347.5, *p* = .023 FA-AU: U = 385.0, *p* = .015
	M2 (commission errors)	0.485	0.508	0.710	0.529	0.543	0.561	χ(2) = 0.690 *p* = 0.707	
	M3 (false negatives)	1.364	1.220	1.194	0.980	1.371	1.114	H(2) = 0.630 *p* = 0.729	
	M4 (omission errors)	28.636	1.220	23.806	0.980	23.629	1.114	H(2) = 67.306 *p* = 2.4e−15*	FA-MG: U = 1020.0, *p* = 3.6e−12 FA-AU: U = 1151.0, *p* = 1.1e−12
H_2_	M5 (severity perception)	0.600	0.641	1.167	1.067	1.297	1.228	H(2) = 6.344 *p* = 0.041*	FA-MG: U = 247.0, *p* = .043 FA-AU: U = 255.0, *p* = .018
	M6 (high severity issues)	0.393	0.432	0.259	0.312	0.252	0.304	H(2) = 1.281 *p* = 0.527	
	M7 (mid. severity issues)	0.457	0.405	0.290	0.318	0.278	0.327	H(2) = 3.363 *p* = 0.186	
	M8 (low severity issues)	0.150	0.238	0.451	0.400	0.470	0.401	H(2) = 11.775 *p* = 0.003*	FA-MG: U = 203.5, *p* = .003 FA-AU: U = 213.0, *p* = .001

The experimental results allowed us to reject H_01_ because a statistical difference emerged for M1 (H(2) = 7.373, *p* = 0.025), as the *full automation* condition was outperformed by both *full augmentation* (U = 385.0, *p* = 0.015) and *middle-ground* (U = 347.5, *p* = 0.023). Similarly, statistical difference emerged for M4 (H(2) = 67.306, *p* = $2.4\times {10}^{-15}$), as in the *full automation* condition participants committed more omission errors than participants in *full augmentation* (U = 1151.0, *p* = $1.1\times {10}^{-12}$) and *middle-ground* (U = 1020.0, *p* = $3.6\times {10}^{-12}$) conditions. No differences were found for M2 (χ(2) = 0.691, *p *= 0.708) and M3 (H(2) = 0.630, *p* = 0.730).

H_02_ can also be rejected because a statistical difference was found for M5 (H(2) = 6.344, *p* = 0.041) and M8 (H(2) = 11.775, *p *= 0.003). In the case of M5, it was found that *full automation* outperformed both *full augmentation* (U = 247.0, *p* = 0.043) and *middle-ground* (U = 255.0, *p* = 0.018). On the contrary, in the case of M8, *full automation* has been outperformed by both *full augmentation* (U = 203.5, *p* = 0.003) and *middle-ground* (U = 203.5, *p* = 0.003). No differences were found for M6 (H(2) = 1.281, *p* = 0.527) and M7 (H(2) = 3.363, *p* = 0.186).

## Discussion and lessons learned

In this study, we investigated whether the level of automation provided by a tool for semi-automatic usability evaluation affects the identification of usability problems. Specifically, we hypothesized that the level of automation does not affect the number of usability problems identified by the evaluators (H_01_) and their perceived severity (H_02_). The results of the study allowed us to reject both hypotheses and derive some lessons learned, highlighted in bold in the following.

For H_01_, it appears that evaluators using **a fully automated approach identify fewer usability problems** than evaluators using the other two approaches proposed in this study (M1). This result reveals a trade-off between the benefits of an automated approach (e.g., less time and expertise required to identify usability problems) and the number of problems identified by evaluators using it. We think that the other two conditions proposed in this study help to discover more usability problems thanks to the visualization of the visitors’ emotions on the heatmaps, which contain more information than the list of problems presented by the *full automation* and push the evaluators to manually inspect the website and the visitors’ emotions. Of course, this result might depend on the list of usability problems reported in the fully automated condition. A longer list could be reported if the threshold for negative emotions is lowered, but this runs the risk of introducing too many false positives. This is an aspect that deserves more attention in future work.

H_01_ also involves the number of false positives identified by the evaluators and shown in the visualization (M2). In this case, it was found that **the detection of false positives was not affected by the level of automation**. This is a rather surprising result because the fully automated approach, which reports the false positive in the list of usability problems and shows it as a usability problem, does not make the evaluators trust it more than the other two conditions, which can benefit from the direct observation of the web page and their reports. In other words, results suggest that commission errors do not depend on the degree of automation, but it is solely the effect of automation bias, due to the knowledge of the presence of an automation^19^.

Another interesting result of H_01_ relates to the number of usability problems discovered by evaluators but not reported in the report (M3). Again, evaluators using **a fully automated approach identify the same number of problems as evaluators of the other conditions**. This result is very positive for the fully automated approach, as it does not limit the evaluators’ ability to go beyond what the report suggests to identify errors that it does not manually. Of course, this requires knowledge of usability problems, so this benefit may not be extended to evaluators without knowledge who wish to benefit from a fully automated approach.

An important result related to H_01_ is in the number of omission errors^19^. It emerged that **a fully automated approach increases the number of omission errors**. In other words, it emerged that participants strongly relied on automation for the detection of usability problems, as a stronger level of automation may obfuscate some. This suggests the need for a human-centered design of AI to ensure safety, and future research may investigate whether the same effect holds in safety–critical domains to inform system design (e.g., medicine).

For H_02_ it resulted that **the severity of the usability problems identified by the evaluators is influenced by the level of automation**. The first indication comes from M5, which suggests that a fully automated approach helps evaluators to perceive and identify the severity of usability problems better than in the other conditions. This is very surprising because the report of usability problems suggested by the full automation does not report the severity, so the evaluators have to analyze the website to interpret the severity of the problem without any other information, as in the case of the two conditions where the heatmap can influence the interpretation. A possible explanation for the lower performance of the *middle-ground* and *full augmentation* approaches could be that the amount of emotion reported by the heatmap leads to a less accurate interpretation of severity. However, this requires further study to confirm.

The final indication for H_02_ comes from the metrics M6, M7, and M8, which quantify the number of usability problems the evaluators found of low, medium, and high severity, respectively. It emerged that **a fully automated approach does not affect the detection of medium- and high-severity errors**, which are the most important ones when evaluating a website. Indeed, the number of low-severity usability problems (M8) detected in the *full automation* condition is significantly lower than in the other conditions. Although this is a negative aspect of *full automation*, it is not confirmed for medium (M7) and high (M6) severity usability problems, where there are no differences between the three experimental conditions.

Summing up, the results of our experiment provide empirical evidence of the importance of the choice of level of automation when designing an AI-based system that aims to automate usability evaluation. Furthermore, the results clearly highlight the need to define evaluators’ goals (e.g., a coarse or deep usability evaluation) to provide them with the most appropriate approach. Specifically, while a *full automation* approach allows for better communication on the severity of the detected problems, a *full augmentation* or *middle-ground* solution allows for better detection of problems in general. Thus, this experiment provides empirical evidence of the importance of human-centered design for AI-based systems, as the specific needs and context of the users should heavily impact the design of the system^18^.

## Threats to validity

We now discuss some aspects that might have threatened the study’s validity to underline under which conditions the study design offers benefits that can be exploited in other contexts, and under which circumstances it might fail.

### Internal validity

It relates to the degree of confidence that the causal relationship tested in this study is not influenced by other factors or variables:

- *Understandability of the material*. Two pilot studies with 5 and 10 participants were executed to validate the understandability of the entire procedure and the material.
- *Expertise of the evaluators*. Since the proposed solutions intend to support evaluators without a strong knowledge of usability evaluation, we recruited students from the third year of the Bachelor of Science in Computer Science degree program who had successfully completed a course on HCI. However, this criterion alone does not guarantee that the evaluator has the minimum knowledge to perform a usability evaluation; thus, we manually screened all the evaluators’ answers to remove participants that gave low-quality answers.
- *Considered usability problems.* Several and different usability problems can be identified while evaluating website usability and they may depend on the specific website. To mitigate this problem, we selected two websites instead of one to increase the heterogeneity of the types of problems. In addition, in the study, participants evaluated a static copy of the website (a still image) to show the evaluators the web pages and the visualisation. Therefore, dynamic elements on the page cannot be fully appreciated and analysed. To limit this problem, we ensured that the selected websites did not have particular usability problems related to dynamic elements.

### External validity

It relates to the possibility of generalizing the study’s findings in different contexts. In this respect, the main threats to our study are as follows:

- *Users’ age.* The participants recruited in our study are mainly under 25 years of age. We can safely accept the experimental results for *digital natives*^34^, but further studies that include older people must be carried out since this aspect limits the generalization of the results.
- *Evaluated websites.* The websites used in this study are not representative of the whole population of websites, which may differ from one another. We mitigated this risk by asking users to evaluate two quite different websites; however, no more than two evaluation sessions could be requested to avoid too long sessions that could reduce participants’ attention. Thus, further studies should be replicated on other websites to observe the extent to which the nature of the website can affect the performance of the degree of automation.
- *Solutions adopted for each level of automation.* To the best of our knowledge, in literature, there are only solutions to automate the discovery of usability problems^9,23^, but no solutions to augment the whole evaluation process exists. This study tries to fill this gap by investigating solutions based on augmentation, representing a first step in this direction. However, further solutions could benefit from other different augmentation techniques, for example, not based on heatmaps as in the case of this research, so the results of this study can be generalized only to those techniques similar to the ones we proposed.
- *Evaluator skills.* Despite the proposed techniques being mainly for evaluators without strong skills, it could be interesting if also experts can benefit from them. This study does not consider them; thus, future work could consider this kind of evaluator to understand the usefulness and acceptance of these techniques by experts.
- *Emotions available in the visualizations.* The current versions of the visualizations considered in this study are based on three emotions, i.e., contempt, joy, and engagement. However, the Ekman model considers other emotions that are not included in the visualization to not overload the participants.

### Conclusion validity

Conclusion validity refers to the validity of the statistical tests applied. In our study, this was alleviated by applying the most common tests employed in HCI and empirical software engineering^35,36^.

## Conclusion and future work

This study contributes to the emerging research area of HCAI threading the line between automation and human control in the context of user studies supported by AI-based systems. The paper highlights the importance of striking a balance between human expertise and AI capabilities to improve website usability evaluation. It emerged that a fully automated approach, which is desirable as it simplifies and speeds up the whole evaluation process, could help evaluators to detect a significant number of usability problems; however, more usability problems can be detected by using one of the augmented approaches proposed in this study. The main difference is that evaluators using a fully automated approach identify fewer low-severity usability problems, but they find the same number of problems of medium and high severity as in the case of augmented solutions. This result suggests that a fully automated solution could be used, for example, to perform a two-steps analysis: the first one ensures the identification of a good number of medium and high severity problems, i.e. the most critical ones on the site; if necessary, the second one could be performed using an augmented technique which could lead to the identification of further problems, in particular low severity ones.

In future work, we plan to extend the exploration to different techniques corresponding to different levels of automation, involving different types of participants (e.g., including experts), evaluating more websites with more usability problems, and investigating how more emotions might affect the different levels of automation. Moreover, we planned to explore if significant differences exist between the usability errors found by experts (e.g., the ground truth in this study) and the ones found by SERENE.

### Supplementary Information

Supplementary Information.

## Data Availability

All the experimental data, i.e., the ground truth (ground-truth.csv), participants’ answers mapped with the ground truth (answers.json), the questionnaires (questionnaires.pdf), and the metrics (metrics.csv) are included in this published article (and its Supplementary Information files).
