# Supplementary material for: The fine line between automation and augmentation in website usability evaluation
Source: Sci Rep. 2024 May 2;14:10129. doi: 10.1038/s41598-024-59616-0 (PMC11066064; doi:10.1038/s41598-024-59616-0)
Supplement: Supplementary file 1 — Supplementary Information. [file 41598_2024_59616_MOESM1_ESM.zip › questionnaire.pdf]

UNIVERSITÀ DEGLI STUDI DI BARI ALDO MORO

SERENE: Semi-Automatic UX Smells Detector

University of Bari "Aldo Moro" Department of Computer Science, IVU Laboratory

Andrea Esposito
andrea.esposito@uniba.it

Prof. Giuseppe Desolda
giuseppe.desolda@uniba.it

Prof. Rosa Lanzilotti
rosa.lanzilotti@uniba.it

Benvenuto!
Ti ringraziamo per aver deciso di prendere parte alla nostra ricerca.

Prima di partecipare, devi registrarti all'esperimento. Ti chiediamo di inserire la tua e-mail. Questo dato non è collegato alle risposte che fornirai nel questionario: ci è utile solo per poterti ricontattare in caso di necessità. Al termine della registrazione, riceverai una chiave personale che potrai utilizzare per accedere al questionario: questa chiave non è collegata alle risposte che fornirai, ma è utilizzata solo per impedire l'invio di risposte multiple.

La tua email saranno trattati nel completo rispetto del regolamento Europeo sulla privacy GDPR. Le tue risposte saranno completamente anonime, come definito dal modulo di consenso informato. Per evitare di influenzare le tue risposte, non possiamo fornire un accesso ai dati prima del completamento del questionario.

E-Mail

name@example.com

☐ Acconsento al trattamento dei miei dati secondo il consenso informato (obbligatorio)

Registrati

UNIVERSITÀ DEGLI STUDI DI BARI ALDO MORO

Grazie per aver deciso di partecipare a questo studio! Ti ricordiamo che tutto il processo di raccolta dati è completamento anonimo.

In questo questionario, ti saranno poste inizialmente delle semplici domande demografiche, per aiutarci a conoscere meglio la composizione del campione di partecipanti. Successivamente, ti saranno mostrate delle immagini estratte dal nostro sistema "SERENE" per il riconoscimento semi-automatico di problemi di usabilità. Per ogni schermata, avrai sulla sinistra la schermata estratta dal sistema e sulla destra ci saranno domande sulla capacità di riconoscere problemi di usabilità osservando la schermata. Se il tuo schermo è troppo piccolo, le due sezioni saranno in un'unica colonna.

Ricorda che non stiamo cercando di valutare la *tua* capacità di riconoscere problemi di usabilità, ma quella del *sistema* di comunicare i problemi che riconosce. Rispondi sinceramente e ricorda che l'intero processo è anonimo, quindi non ci saranno premi o punizioni per risposte rispettivamente giuste e sbagliate.

Quando sei pronto, vai avanti per iniziare il questionario.

Indietro

Avanti

UNIVERSITÀ DEGLI STUDI DI BARI ALDO MORO

Anagrafica

Età *

23

Genere *

Maschio

Quanto ore usi mediamente internet ogni giorno? *

Da 13 a 18 ore

Indietro

Avanti

Valutazione Problemi di Usabilità: Comune di Taranto

Mostra le euristiche di Nielsen

Dati mostrati:

☑ Mostra il sito

Ricorda che puoi cambiare la visualizzazione usando le opzioni qui sopra

Report: potenziali problemi

- Problema grave in: "navbar" (alta concentrazione di: disprezzo)
- Problema in: "sezione Link" (media concentrazione di: disprezzo)
- Problema in: "sezione Gallery" (media concentrazione di: disprezzo)

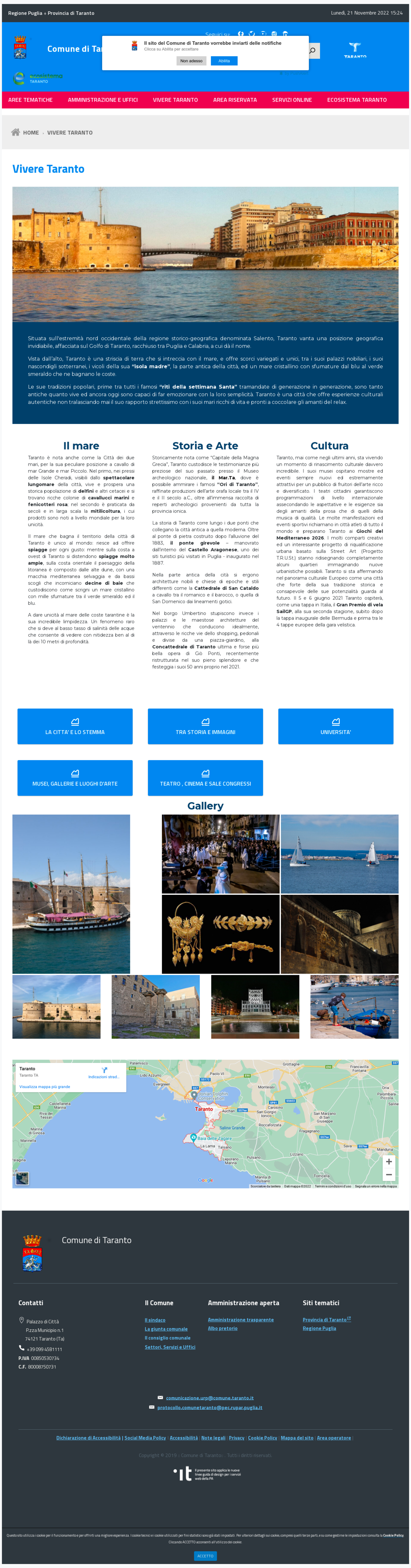

Riesci a riconoscere qualche problema di usabilità dal report osservando il sito? *

◉ Sì ○ No

Quali sono i problemi di usabilità che riconosci? Elencali qui sotto, specificando per ciascuno di essi: elemento dell'interfaccia affetto dal problema, tipo di problema e gravità (1-3, 3 massima gravità). *

Elemento UI	Problema	Gravità
Home	Overlay	3
		1

Riconosci altri problemi di usabilità che non sono evidenziati nel report? *

◉ Sì ○ No

Quali sono i problemi di usabilità che il report non evidenzia? Elencali qui sotto, specificando per ciascuno di essi: elemento dell'interfaccia affetto dal problema, tipo di problema e gravità (1-3, 3 massima gravità). *

Elemento UI	Problema	Gravità
Home	Troppi colori	1
		1

Indietro

Avanti

UNIVERSITÀ DEGLI STUDI DI BARI ALDO MORO

Valutazione Problemi di Usabilità: Regione Basilicata

Mostra le euristiche di Nielsen

Dati mostrati:

☑ Mostra il sito

Ricorda che puoi cambiare la visualizzazione usando le opzioni qui sopra

Report: potenziali problemi

- Problema grave in: "sezione Avvisi" (alta concentrazione di: contempt)
- Problema in: "sezione Link" (bassa concentrazione di: coinvolgimento)

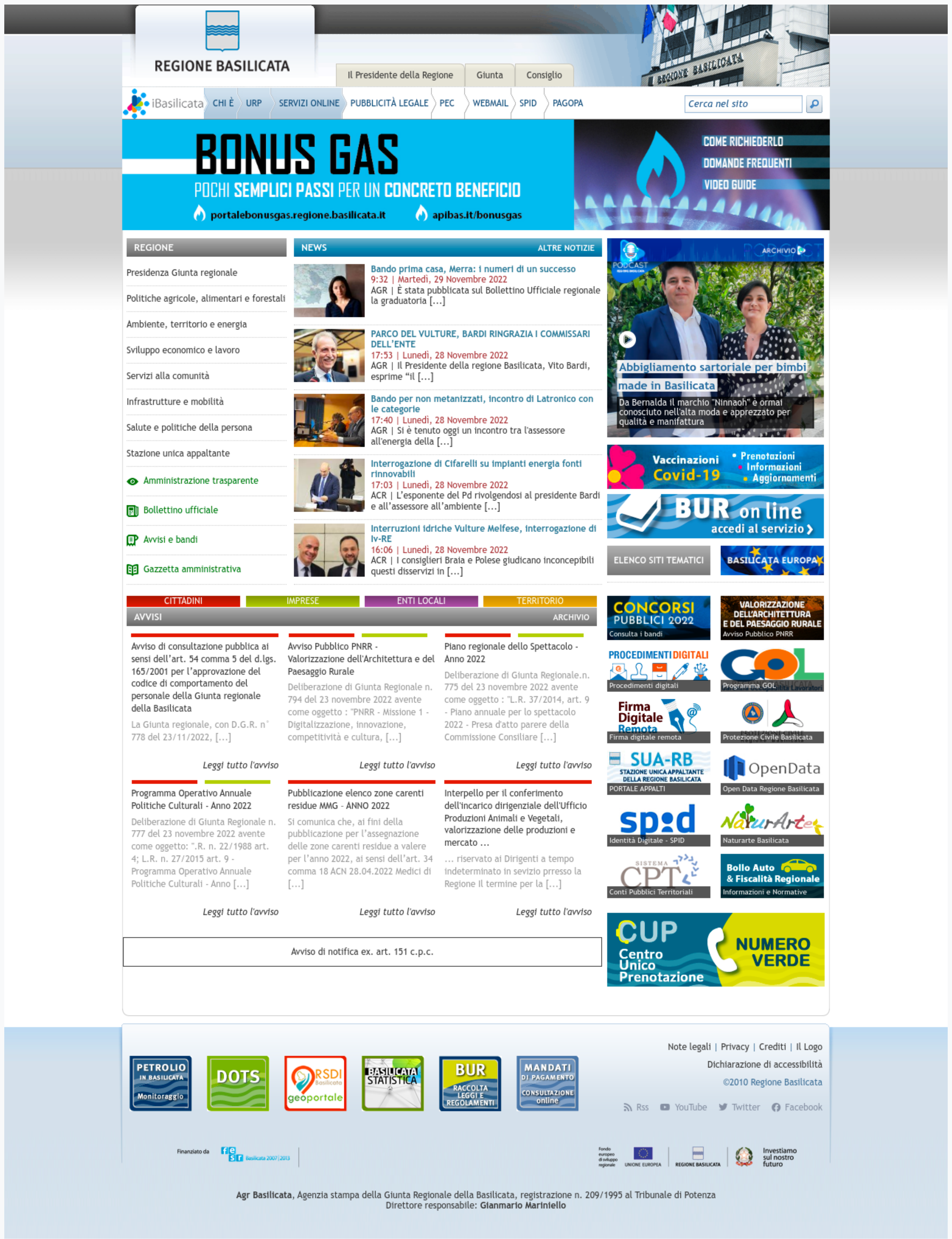

Riesci a riconoscere qualche problema di usabilità dal report osservando il sito? *

(•) Sì ( ) No

Quali sono i problemi di usabilità che riconosci? Elencali qui sotto, specificando per ciascuno di essi: elemento dell'interfaccia affetto dal problema, tipo di problema e gravità (1-3, 3 massima gravità). *

Elemento UI	Problema	Gravità
Home	Confusa	3
		1

Riconosci altri problemi di usabilità che non sono evidenziati nel report? *

( ) Sì (•) No

Quali sono i problemi di usabilità che il report non evidenzia? Elencali qui sotto, specificando per ciascuno di essi: elemento dell'interfaccia affetto dal problema, tipo di problema e gravità (1-3, 3 massima gravità).

Elemento UI	Problema	Gravità
		1

Indietro

Invia
